# Supplementary material for: Profiling Glioblastoma Cases with an Expression of DCX, OLIG2 and NES
Source: Int J Mol Sci. 2021 Dec 8;22(24):13217. doi: 10.3390/ijms222413217 (PMC8708973; doi:10.3390/ijms222413217)
Supplement: Supplementary file 1 [file ijms-22-13217-s001.zip › ijms-1445747-supplementary.pdf]

International Journal of
Molecular Sciences

MDPI

Supplementary materials

Profiling Glioblastoma Cases with an Expression of DCX, OLIG2 and NES

Supplementary Materials:

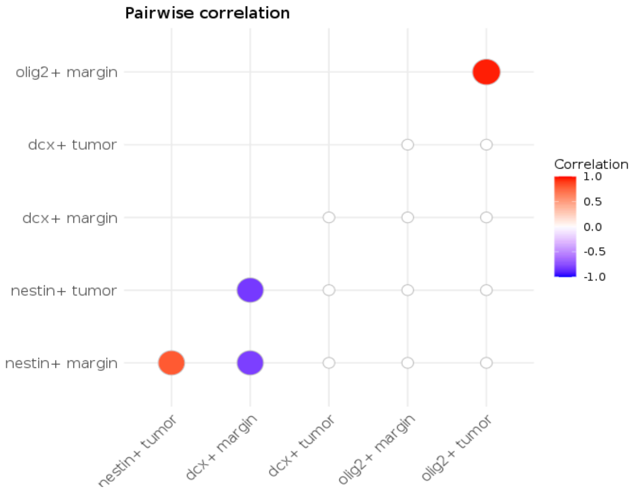

Figure S1. Pairwise correlation of margins and tumor sites adjacent to margins.

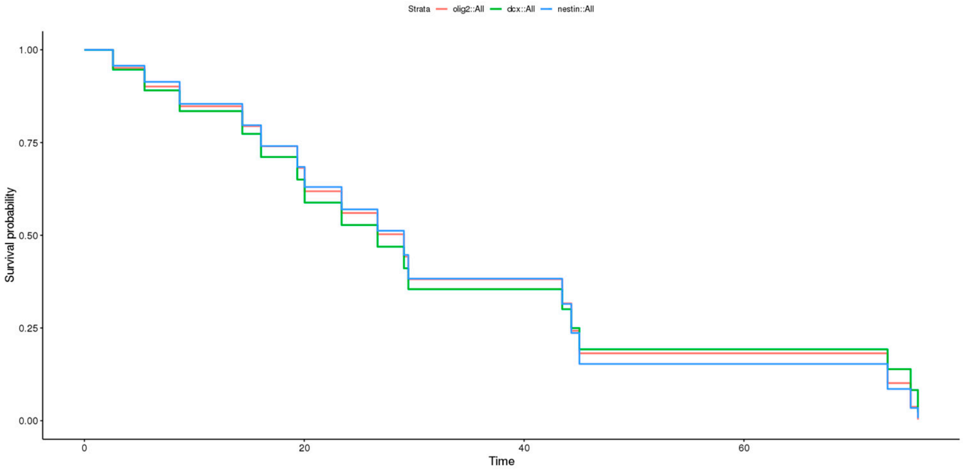

Figure S2. Survival changes concerning the frequency of studied antigens-positive cells.

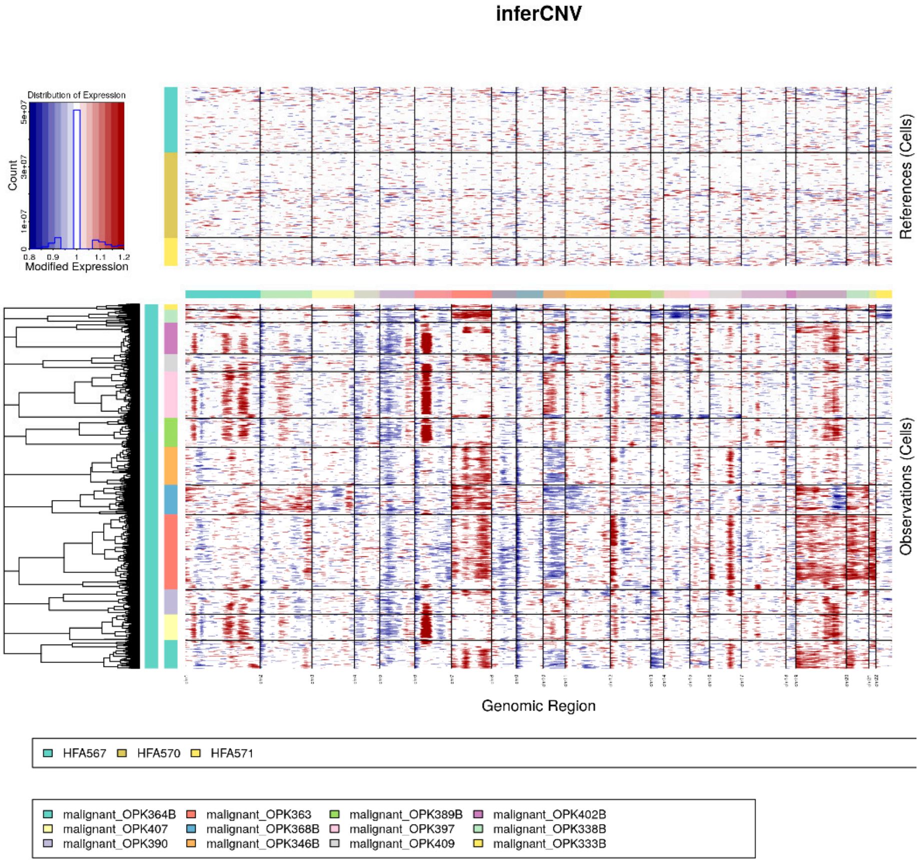

Figure S3. Predicted Copy Number Variations among the single cells from the malignant and fetal origin. The clustered score of genomic regions duplications (red) or loss (blue) had been shown.

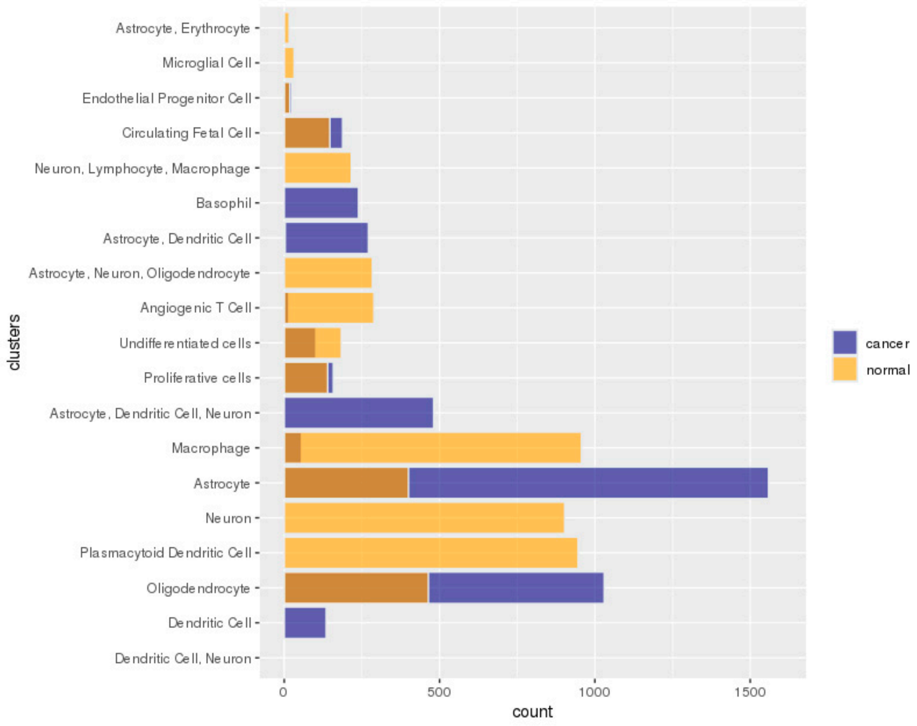

Figure S4. The histogram shows the cells' distribution into the clusters according to the 'cancer' and 'normal' annotations.
